# Dietary profile of patients with Stargardt’s disease and Retinitis Pigmentosa: is there a role for a nutritional approach?

**DOI:** 10.1186/s12886-016-0187-3

**Published:** 2016-01-22

**Authors:** Francesco Sofi, Andrea Sodi, Fabrizio Franco, Vittoria Murro, Dania Biagini, Alba Miele, Giacomo Abbruzzese, Dario Pasquale Mucciolo, Gianni Virgili, Ugo Menchini, Alessandro Casini, Stanislao Rizzo

**Affiliations:** Department of Experimental and Clinical Medicine, University of Florence, Florence, Italy; Agency of Nutrition, University Hospital of Careggi, Florence, Italy; Don Carlo Gnocchi Foundation Italy, Onlus IRCCS, Florence, Italy; Department of Surgery and Translational Medicine, Eye Clinic, University of Florence, Florence, Italy

**Keywords:** Stargardt’s disease, Retinitis Pigmentosa, Vitamin A, Diet

## Abstract

**Background:**

Stargardt’s disease (STGD) and Retinitis Pigmentosa (RP) are inherited retinal degenerations that may be affected, in opposite way, by diet.

**Methods:**

Dietary profile was assessed in 24 patients with STGD and in 56 patients with RP. We documented in only 6 out of 24 (25 %) STGD patients a daily intake of vitamin A within the recommended range while 14/24 (58.3 %) reported a high daily intake and 4/24 (16.7 %) showed a low daily intake. With regard to RP, 4/56 (7.1 %) reported to be within the recommended range, 37/56 (66.1 %) reported high daily intake and 15/56 (26.8 %) showed low daily intake of vitamin A.

**Results:**

Interestingly, STGD patients with low vitamin A intake (<600 µg RAE/day) showed significantly better visual acuity with respect to those introducing higher intake of vitamin A.

**Conclusion:**

The present study suggests insuitable nutrient intakes among patients with STGD and RP, especially for daily intake of vitamin A. The results may be used to provide tailored nutritional interventions in these patients.

## Background

Vitamin A plays a crucial role in the biochemistry of visual signal cascade and the maintenance of an optimal vitamin A status has been considered relevant for a normal retinal physiology [1, 2].

Stargardt’s Disease (STGD) and Retinitis Pigmentosa (RP) are genetic-based relevant ocular diseases that may be affected, in opposite way, by vitamin A intake and other nutrients [3–5].

In an animal model of STGD vitamin A supplementation has been shown to accelerate the accumulation of toxic by-products, being prevented by a reduction of its serum levels with possible implications for the treatment [6]. Moreover, in such patients a supplementation with lutein, a retinal carotenoid, increases macula pigment density but it seems not to be associated with changes in central vision over a 6 months-follow up period [7]. Conversely, RP patients seems to get beneficial effects from a supplementation with vitamin A, since it has been associated with an improved preservation of cone electroretinogram amplitudes and has been proposed as a treatment to slow the progression of the disease [8]. Moreover, for RP patients assuming vitamin A therapy addition of the polyunsaturated fatty acid docosahexaenoic acid has been showed to slow the course of the disease over the first two years of supplementation [9–11]. Similarly, an increased dietary intake of lutein, a retinal carotenoid, seems to slow visual function loss in RP adult patients assuming vitamin A [12]. In the light of all these clinical observations, nutritional indications and/or supplementations for patients suffering from these retinal dystrophies are usually given but, to the best of our knowledge, no data on dietary habits of these patients are presently available.

The purpose of this study was to evaluate the dietary habits and nutritional intake of vitamin A in patients with STGD and RP, in order to suggest tailored dietary modifications in such diseases.

## Methods

### Study population

The study populations comprised 24 patients with a STGD [12 M, 12 F; median age: 34 years (range: 13–64)] and 56 patients with RP [23 M, 33 F; median age: 45 years (range: 14–85)] referring to the Eye Clinic of the University of Florence, Italy for clinical evaluation. For patients under 18 years of age, the informed consent was obtained from their parents.

The criteria for STGD phenotype included the following: appearance in the first or second decade of life; bilateral progressive central vision loss; macular atrophy/dystrophy; normal calibre of retinal vessels; absence of pigmented bone spicules; and normal or mildly abnormal full-field electroretinogram. Flecks at the posterior pole and dark choroid at fluorescein angiography were present in most of the cases but their absence was not considered an exclusion criterion. All the patients carried bi-allelic mutations of the ABCA4 gene [13, 14]. The clinical diagnosis of RP was based on the history of night blindness, typical retinal pigmentary changes, attenuated retinal vessels, reduced or absent electroretinogram response and progressive peripheral visual field loss. For these patients the molecular analysis could not be performed.

**Fig. 1:**
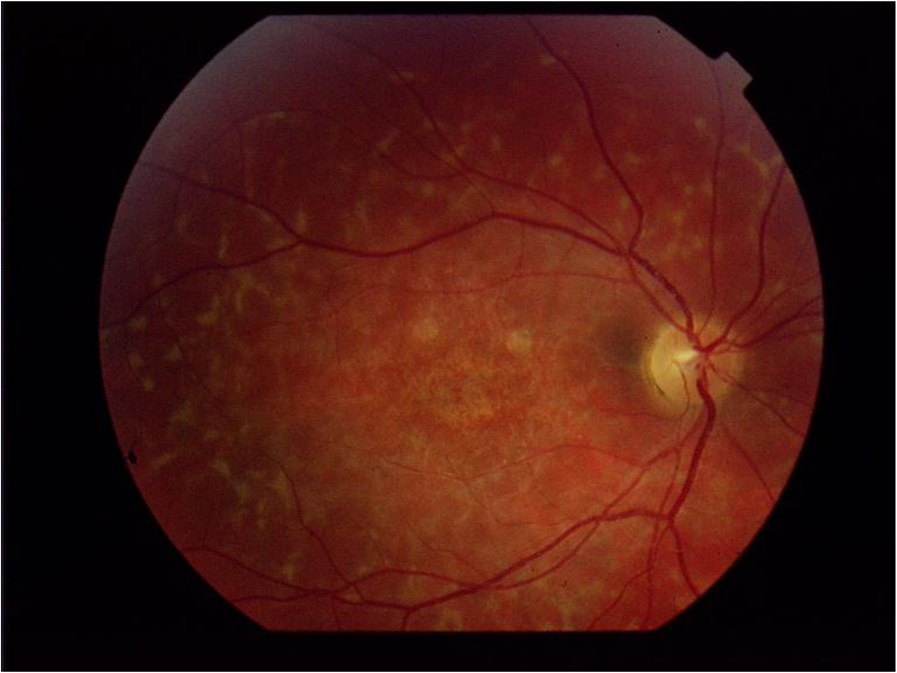
STGD: macular atrophy and flecks at the posterior pole

**Fig. 2:**
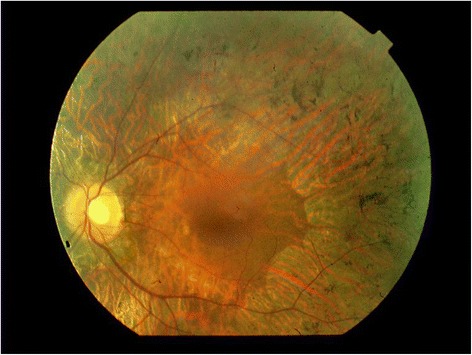
RP: Pale optic disc, attenuated retinal vessels, midperipheral dystrophy of the retinal pigment epithelium with typical pigmentary changes

Figure 1 illustrates a typical STGD while Fig. 2 shows the fundus appearance of a classic RP.

All the patients included in the study were clinically evaluated by means of a standard ophthalmologic examination, electroretinography (Electrophysiological Diagnostic Unit Retimax, Roland Consult, Brandenburg, Germany) performed according to the existing ISCEV Guidelines, OCT scan (Topcon 3D OCT-1000, Topcon Medical Systems Inc, Oakland, NJ, USA) and automated visual field examination (Humprey Automated Perimeter, Carl Zeiss, Dublin, CA, USA). Fluorescein angiography (Zeiss Retinograph with Image Processing Software Visupac, Carl Zeiss, Dublin, CA, USA) was obtained in 15 STGD patients either to refine the diagnosis or to investigate the possible presence of dark choroid.

In both groups age of onset of the visual symptoms and visual acuity at the time of the dietary analysis were considered as possible markers of disease severity. The study adhered to the tenets of the Declaration of Helsinki and was approved by the AOU Careggi Ethics Committee. Each patient gave written informed consent.

### Dietary analysis

Trained dieticians collected data in order to assess the habitual consumption of 109 food items, with the aid of software specific for the analysis of food habits and the estimation of nutrient and caloric intake (WinFood, release 1.5; Medimatica, Martinsicuro, Te, Italy). For each specific food item a commonly used portion size was specified and subjects were asked how often they had consumed that unit (never, daily, weekly, monthly) on average during the past year. Emphasis was paid to ensure that the answers were related to a yearlong dietary pattern and not to last few months, especially in terms of seasonal changes of diet. Nutrient intakes were presented for comparison with the dietary reference intakes (RDI) for males and females aged 18–59 years [15].

### Statistical analysis

Statistical analysis was performed by using the SPSS (Statistical Package for Social Sciences Inc., Chicago, IL, USA) software for Macintosh (Version 19.0). Values are expressed as mean ± standard deviation (SD) or median and range, as appropriate. Chi-square test was used to test for proportions. T-test was used for comparisons between single groups for normally distributed parameters and Mann–Whitney test was used for comparison of age between groups. A *p*-value <0.05 was considered to indicate statistical significance.

## Results

### Clinical characteristics

The median age at onset of symptoms was 20.5 years (range: 8–39) for STGD and 30 years (range: 2–72) for RP, with the median duration of the disease of 11.5 years (range: 0–39) and 14 years (range: 0–53) for STGD and RP, respectively.

At the moment of the dietary analysis Snellen equivalent visual acuity was 3.8 ± 3.1 in the right eye and 3.8 ± 3.2 in the left eye for STGD as compared to 5.4 ± 3.8 in the right eye and 5.1 ± 3.7 in the left eye for RP patients.

### Dietary profile

Dietary pattern of the study population is described in Tables 1 and 2, for STGD and RP respectively. Macronutrient distributions were not significantly different between males and females for both groups of patients. By comparing the dietary patterns with the dietary reference intakes reported by the Italian Society of Human Nutrition (Italian Society of Human Nutrition, [15]) in order to prevent the major chronic diseases we can observe some divergences from the recommendations. Indeed, in both males and females the study populations showed a slightly increased contribution of total fat from the diet, with a considerably low contribution from polyunsaturated fats. In addition, a low intake of fibre is showed in both genders of the two groups of patients as well as a high intake of cholesterol was demonstrated in all the patients.

**Table 1:** Dietary profile and nutrient intake in patients with Stargardt’s Disease

	Males (*n* = 12)		Females (*n* = 12)	
	Mean (SD)	DRI	Mean (SD)	DRI
Energy, kcal	2249.9 (309.4)	2200	1773 (338.2)	1800
% of energy from carbohydrates	51.3 (4.4)	45–60 %	52.4 (7.9)	45–60 %
% of energy from protein	15.6 (1.8)	15–20 %	15.8 (2)	15–20 %
% of energy from fats	33.2 (5)	<30 %	34.8 (7.3)	<30 %
% of energy from saturated fats	8.7 (2.3)	<10 %	9.8 (3)	< 10 %
% of energy from monounsaturated fats	15.3 (2.7)	15–20 %	14.9 (4.7)	15–20 %
% of energy from polyunsaturated fats	3.4 (0.6)	5–10 %	3.5 (0.9)	5–10 %
EPA, mg	230 (130)	>250	120 (70)	>250
DHA, mg	290 (70)		130 (90)	
Cholesterol, mg	286.7 (104.8)	<200	209.5 (56.9)	<200
Dietary fibre, g	18.5 (4.3)	25	16.3 (4.6)	25
Calcium, mg	707.3 (250.6)	1000	615.9 (220.8)	1000
Magnesium, mg	146.4 (32.1)	240	130.9 (26.3)	240
Potassium, mg	2394.8 (767.1)	3900	2225.3 (495.5)	3900
Sodium, mg	1462.3 (606)	1500	1476.2 (832.2)	1500
Iron, mg	10.2 (1.7)	10	13.5 (2.3)	18
Vitamin A, μg RAE	1277.9 (962.8)	700	729.4 (258.8)	600
Thiamin, mg	1.23 (0.25)	1.2	1.19 (0.32)	1.1
Riboflavin, mg	1.56 (0.47)	1.6	1.16 (0.32)	1.3
Niacin, mg	16.9 (2.4)	18	13.9 (2.9)	18
Vitamin B6, mg	1.14 (0.43)	1.3	1.17 (0.53)	1.3
Folic acid, mg	193.2 (100.3)	400	201.5 (80.9)	400
Vitamin C, mg	161.7 (103.6)	105	136.2 (73.8)	85
Vitamin D, μg	3.41 (1.71)	15	2.33 (1.65)	15
Vitamin E, mg	11.1 (3.5)	15	7.92 (3.7)	15

The dietary reference intakes (DRI) are presented using adequate intake and population reference intake for males and females aged 18–59 years according to the Dietary Reference Values of the Italian Society of Human Nutrition [15]

*EPA* eicosapentaenoic acid, *DHA* docosahexaenoic acid, *RAE* retinol activity equivalent

**Table 2:** Dietary profile and nutrient intake in patients with Retinitis Pigmentosa

	Males (*n* = 23)		Females (*n* = 33)	
	Mean (SD)	DRI	Mean (SD)	DRI
Energy, kcal	1994.9 (436.4)	2200	1830 (362.1)	1800
% of energy from carbohydrates	47.9 (7.9)	45–60 %	49.6 (6.2)	45–60 %
% of energy from protein	16.7 (2.6)	15–20 %	17.7 (2.8)	15–20 %
% of energy from fats	34.6 (6.2)	<30 %	34.1 (5.6)	<30 %
% of energy from saturated fats	9.3 (2.8)	< 10 %	9.3 (2.3)	< 10 %
% of energy from monounsaturated fats	15.6 (3.6)	15–20 %	15 (3.7)	15–20 %
% of energy from polyunsaturated fats	3.6 (1)	5–10 %	3.4 (0.9)	5–10 %
EPA, mg	180 (130)	>250	230 (60)	>250
DHA, mg	190 (150)		280 (60)	
Cholesterol, mg	284.4 (113.9)	<200	266.3 (87.6)	<200
Dietary fibre, g	15.5 (4.5)	25	15.9 (4.3)	25
Calcium, mg	596.9 (198.8)	1000	662.5 (213.9)	1000
Magnesium, mg	138.8 (50.4)	240	139.6 (33.6)	240
Potassium, mg	2432.8 (511.5)	3900	2423.9 (552.5)	3900
Sodium, mg	1626.8 (563.1)	1500	1357.8 (443.1)	1500
Iron, mg	11.7 (3.7)	10	10.9 (2.8)	18
Vitamin A, μg RAE	1332.9 (205.7)	700	1368.9 (498.7)	600
Thiamin, mg	1.07 (0.35)	1.2	1.05 (0.21)	1.1
Riboflavin, mg	1.56 (0.47)	1.6	1.16 (0.32)	1.3
Niacin, mg	17.3 (5.4)	18	15.4 (4.9)	18
Vitamin B6, mg	1.26 (0.43)	1.3	1.33 (0.39)	1.3
Folic acid, mg	203.9 (80.4)	400	221.2 (66.8)	400
Vitamin C, mg	112.1 (60.6)	105	136.3 (50.5)	85
Vitamin D, μg	3.83 (2.76)	15	4.44 (2.24)	15
Vitamin E, mg	9.7 (3.6)	15	9.2 (3.1)	15

The dietary reference intakes (DRI) are presented using adequate intake and population reference intake for males and females aged 18–59 years according to the Dietary Reference Values of the Italian Society of Human Nutrition [15]

*EPA* eicosapentaenoic acid, *DHA* docosahexaenoic acid, *RAE* retinol activity equivalent

As considering minerals and vitamins, both groups of patients reported insufficient intake of some minerals and vitamins, as showed by low intake of calcium, magnesium, potassium, vitamin B6, folic acid and vitamin D.

In Table 3, prevalence of patients following recommendations for a healthy diet is reported. Notably, a high proportion of patients consume a hyperlipidic diet with a contribution from total fat >30 % of the total energy in 19/24 (79.2 %) STGD and 45/56 (80.4 %) RP patients. More importantly, almost the total number of patients enrolled in the study showed an insufficient intake for polyunsaturated fats, and a high proportion of the patients reported a high intake of cholesterol from the diet. Similarly, almost the totality of the patients did not reach current recommendations for fibre intake.

**Table 3:** Prevalence of men and women following the dietary reference intake (DRI) for selected nutrients

	Stargardt’s disease		Retinitis pigmentosa	
	Males (*n* = 12)	Females (*n* = 12)	Males (*n* = 23)	Females (*n* = 33)
Carbohydrates (45–60 % TE), *n* (%)	11 (91.7)	7 (58.3)	13 (56.5)	23 (69.7)
Protein (15–20 % TE), *n* (%)	7 (58.3)	7 (58.3)	14 (60.9)	22 (66.7)
Fats (<30 % TE), *n* (%)	2 (16.7)	3 (25)	5 (21.7)	6 (18.7)
SFA (<10 % of total fats), *n* (%)	8 (66.7)	7 (58.3)	15 (65.2)	19 (57.6)
MUFA (15–20 % of total fats), *n* (%)	8 (66.7)	5 (41.7)	10 (43.5)	15 (45.5)
PUFA (5–10 % of total fats), *n* (%)	0 (0)	0 (0)	1 (4.3)	0 (0)
Cholesterol (<200 mg), *n* (%)	3 (25)	6 (50)	5 (21.7)	8 (24.2)
Dietary fibre (>25 g), *n* (%)	0 (0)	0 (0)	0 (0)	1 (3)
Vitamin A (600–700 μg RAE), *n* (%)	1 (8.3)	5 (41.7)	2 (8.7)	2 (6.1)
EPA + DHA (>250 mg), *n* (%)	10 (83.3)	4 (33.3)	15 (65.2)	24 (72.7)

DRIs for the selected macronutrient or nutrient are reported in brackets

*STGD* Stargardt’s Disease, *PR* pigmentosa retinitis, *TE* total energy, *SFA* saturated fats, *MUFA* monounsaturated fats, *PUFA* polyunstaurated fats, *EPA* eicosapentaenoic acid, *RAE* retinal activity equivalent, *DHA* docosahexaenoic acid

### Vitamin A and other nutrients

**Fig. 3:**
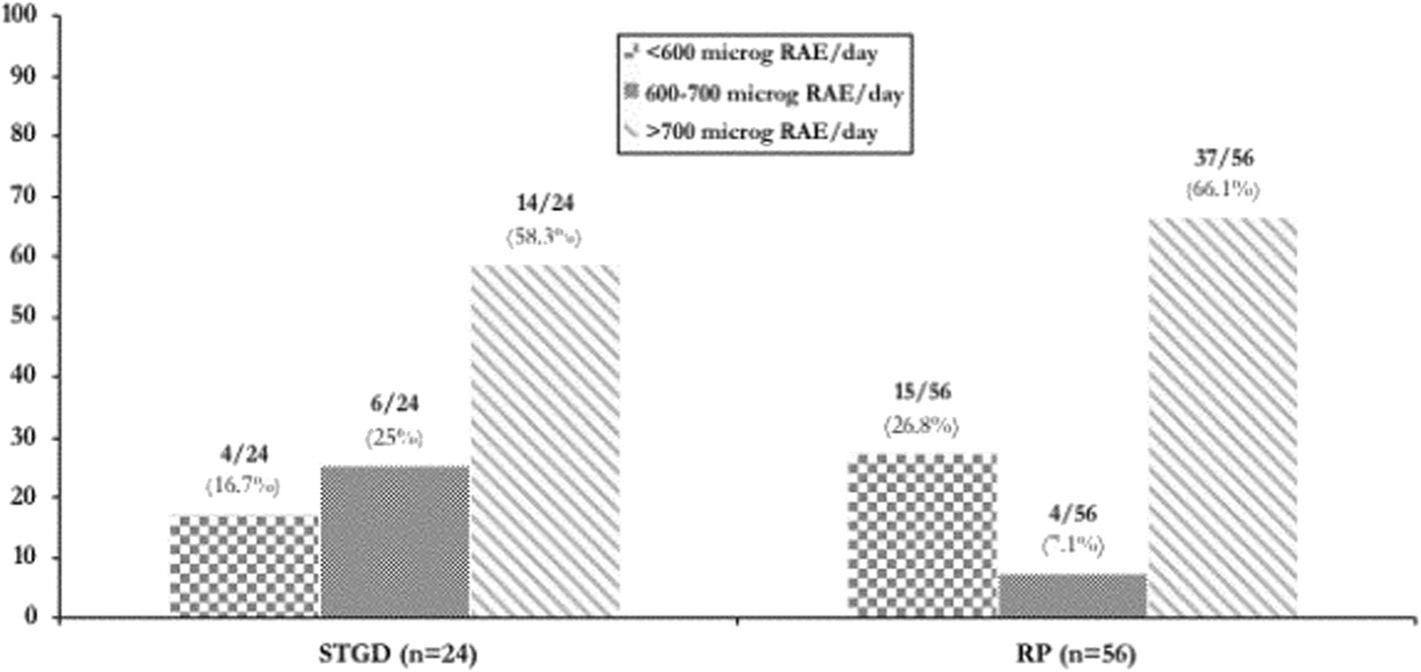
Prevalence of STGD and RP patients according to dietary recommendations of vitamin A

With regard to vitamin A intake, only 6 out of 24 STGD patients (25 %) reported a daily intake of vitamin A that follows the recommendations for a healthy diet (Table 3 and Fig. 3). Contrarily to what suggested for patients with such disease, interestingly, in 14/24 (58.3 %) a high intake of vitamin A from the diet was reported. In this group of patients the average intake of dietary vitamin A was 1329.8 µg RAE/day (SD: 828.4) with a range of 706.3–3863.2 µg RAE/day, and one patient exceeded the upper toxicity level (3000 µg RAE/day). Looking at the clinical characteristics, it came evident that such patient presented with a very low visual acuity (right eye: 1.0; left eye: 1.2) and an early age of onset of the disease (19 years). Similarly, as far as RP is concerned, only 4 out of 56 patients (7.1 %) reported to meet the current recommendations for daily intake of vitamin A from the diet. Even in this group of patients, contrarily to what usually recommended by physicians, a relatively high proportion of patients, i.e., 15/56 (26.8 %) reported to consume a low intake of vitamin A (<600 µg RAE/day) (Fig. 3).

Interestingly, STGD patients with low vitamin A intake (<600 µg RAE/day) showed significantly better visual acuity (right eye: 6.8 ± 3.4; left eye: 7.0 ± 3.8 vs. 3.2 ± 2.8 and 3.2 ± 2.7; p = 0.04) with respect to those introducing higher intake of vitamin A (>700 µg RAE/day). On the other hand, RP patients with high vitamin A intake (>700 µg RAE/day) showed a higher, albeit not significant, age of onset of the disease (30.8 ± 5.7 years) vs. those reporting low vitamin A intake (25.5 ± 9.0 years).

Finally, intake of EPA and DHA from diet was also evaluated. Among STGD patients, 10 out of 24 (41.7 %) patients reported not to reach the recommendations for these relevant nutrients (>250 mg/day). A similar result has been reported in the RP group since 17 out of 56 (30.4 %) patients showed not to reach the current recommendations for the introduction of EPA + DHA with the diet.

## Discussion

Over the last years, some nutrients like vitamin A, docosahexaenoic acid, and lutein have been proposed to influence the clinical course of the two most common retinal dystrophies, STGD and RP [8, 9, 12]. In fact in STGD vitamin A cannot be properly metabolized because of ABCA4 protein impairment and gives origin to toxic byproducts which are the main components of lipofuscin [2, 6]. On the other side in most RP patients genetic abnormalities prevent the appropriate processing of vitamin A in phototransduction and visual cycle [2, 4, 5, 16]. Indeed, in opposite ways, both STGD and RP may be significantly influenced by dietary intake of these nutrients.

In STGD, experimental studies in animal models suggested that a reduction of vitamin A dietary intake, and their resulting circulating levels, might arrest the accumulation of retinal toxic metabolites, so ameliorating the clinical course of the disease [17]. However, a low vitamin A diet has never been proposed and evaluation of dietary profiles of these patients has never been conducted. Conversely, in RP, nutritional supplementation with vitamin A associated with docosahexaenoic acid or lutein has been already evaluated in clinical trials [8, 10, 12], showing some beneficial effects, even if the efficacy and safety of this approach is still controversial: in fact, the high doses of vitamin A proposed in RP patients have the potential risk of bone and liver damage, and severe birth defects if assumed by pregnant women [18].

In our study we investigated the dietary habits of some patients affected by RP or STGD in order to seek for possible tailored dietary intervention to suggest to these patients. The study population showed some divergences from nutritional recommendations that may be corrected through a nutritional intervention. Indeed, a high proportion of patients consume a hyperlipidic diet with a high assumption of cholesterol and a low intake of polyunsaturated fatty acids and fibre. Interestingly, with regard to vitamin A intake, we reported that the daily intake of vitamin A in STGD patients was higher than what recommended in a relevant portion of the study population. In this group of patients, the high intake of vitamin A was also associated with a worst clinical course of the disease in terms of visual acuity. Conversely, in RP patients vitamin A intake was lower than dietary recommendations in a relevant percentage of RP patients, with a resultant - even if not significant - worst clinical profile in terms of earlier age of onset of the disease. These data, showing a relevant prevalence of insuitable nutrient intakes among our patients with retinal dystrophies, supports the possible influence of diet on the pathogenesis of some inherited retinal degenerations and suggests a nutritional intervention in these patients.

Actually, the dietary pattern of these patients may play a significant role in the clinical course of the disease and may represent a therapeutic approach for disorders presently without effective treatment.

The study has some limitations. First, the small sample size of the study population does not allow us to establish possible relationships between nutrients and different clinical features of the retinal degenerations. On the other hand, it should be noted that STGD is a relatively rare condition and this is the first study investigating this issue. Second, the nature of the study i.e., cross-sectional design does not give us the opportunity to confirm a causal relationship between nutritional and clinical characteristics of these diseases. Moreover RP and STGD may show a very high phenotypic variability and so the association between nutrients intake and clinical course must be considered not conclusive. Third, circulating levels of vitamin A could not be measured in such patients. The measurements of serum vitamin A levels would have improved the knowledge of the pathophysiological mechanisms at the basis of the onset and of the grade of severity of the disease. Finally the possible influence of multiple lifestyle features (like smoking, drugs assumption, BMI) should be considered in future investigations.

## Conclusions

Nevertheless, despite all these limitations, the strength of the present study is that it is the first study that evaluated the dietary profile of patients with retinal dystrophies. The altered intake of many nutrients in the diet of these patients supports the idea that a nutritional intervention can be attempted before giving a prescription with nutritional supplementations. Further studies are requested to evaluate the balance between the possible clinical benefits of a reduced vitamin A intake and the potential ocular (night blindness and xerophthalmia) and systemic (depression, skin problems and others) risk of vitamin A deficiency.

## Abbreviations

RAE: retinal activity equivalent
RP: Retinitis Pigmentosa
STGD: Stargardt’s disease
